# Access to anticancer medicines in public hospitals of Northwestern China

**DOI:** 10.3389/fpubh.2023.1182617

**Published:** 2023-05-19

**Authors:** Yue Ma, Jin Peng, Xuelin Yao, Liuxin Feng, Xinke Shi, Minghuan Jiang

**Affiliations:** ^1^Department of Pharmacy Administration and Clinical Pharmacy, School of Pharmacy, Xi’an Jiaotong University, Xi’an, China; ^2^Center for Drug Safety and Policy Research, Xi’an Jiaotong University, Xi’an, China; ^3^Shaanxi Center for Health Reform and Development Research, Xi’an, China; ^4^Research Institute for Drug Safety and Monitoring, Institute of Pharmaceutical Science and Technology, Western China Science and Technology Innovation Harbor, Xi’an, China; ^5^Department of Pharmacy, The Second Affiliated Hospital of Xi’an Jiaotong University, Xi’an, China; ^6^Health Science Center, Xi’an Jiaotong University, Xi’an, China

**Keywords:** anticancer medicine, availability, defined daily dose, affordability, public hospital

## Abstract

**Objective:**

We aimed to evaluate the accessibility of anticancer medicines in public hospitals of Shaanxi, a representative province of Northwestern China.

**Methods:**

Thirty-one anticancer medicines were investigated in 146 designated public hospitals in 10 cities of Shaanxi Province. We used medicine procurement data from the Shaanxi Drug Centralized Purchasing Platform during 2019–2021. Primary outcomes included the availability, drug utilization, and affordability of anticancer medicines.

**Results:**

The mean availability of 31 anticancer medicines increased significantly from 5.45% in 2019 to 14.72% in 2021. The mean availability of nationally negotiated medicines was significantly lower than that of Class B medicines (8.72% vs. 12.85%, *p* = 0.048), whilst the availability of injectable medicines was significantly greater than that of oral medicines (13.66% vs. 8.77%, *p* = 0.007). In 2019–2021, the annual mean amount purchased increased significantly from CNY 6.51 million to CNY 18.56 million (*p* = 0.007). The mean defined daily doses of 31 medicines significantly rose from 225.50 to 1019.50 (*p* = 0.008) whereas their defined daily drug cost significantly decreased from CNY 551.15 to CNY 404.50 (*p* < 0.001). The percentage of catastrophic health expenditure decreased from 71.0 to 51.65% and from 90.30 to 80.60% for urban and rural residents, respectively. The affordability of nationally negotiated medicines was significantly lower than that of Class B medicines (*p* = 0.032), and the affordability of injectable medicines had no significant difference compared to that of oral medicines (*p* = 0.124) for both urban and rural residents.

**Conclusion:**

The accessibility of anticancer medicines improved dramatically in public hospitals of Northwestern China during the period 2019–2021.

## 1. Introduction

Cancer is a disease that seriously threatens human health. The number of global cancer cases and deaths increased from 2006 to 2016 by 38 and 17.8%, respectively (1). It is estimated that 28.4 million new cancer cases will occur in 2040, a 47% increase over the number in 2020 (2). In China, there were 4.57 million new cancer cases and 3 million cancer deaths in 2020, accounting for 23.7 and 30.2% of those worldwide, respectively (3). However, the 5-year survival rate of cancer cases in China was lower than that in developed countries. For instance, the 5-year survival rate of breast cancer in China was more than 8% lower than that in the United States and Australia (4).

Access to anticancer medicines is a pivotal part of cancer treatment and influences the survival rate of cancer patients worldwide. Mainly owing to the high cost of anticancer medicines, their accessibility is unsatisfactory in both developed and developing countries (5). Previous studies have reported that the out-of-pocket (OOP) costs for cancer treatment in low- and lower middle-income countries account for 58 and 32% of total costs, respectively, but only 1.8% in upper middle-income countries (6). In Europe, only 66% of surveyed anticancer medicines are always available in at least 90% of countries (7).

The number of newly approved anticancer medicines in China has increased substantially since 2005 (8), with 63.4% (*n* = 52) of these approved during 2017–2020 (9). However, in terms of access to anticancer drugs, China has among the lowest accessibility in the world (10), and only 6 of 49 innovative anticancer medicines newly approved worldwide were available in China during 2010–2014 (11). To improve accessibility, the Chinese government has taken a series of measures, including imposing tariffs on imported cancer drugs exempted in 2018, initiating centralized government negotiations and procurement of anticancer medicines, and incorporating more anticancer drugs into the National Reimbursement Drug List (NRDL) (10).

The high OOP cost, sluggish market approval of new drugs, inadequate funding of public hospitals, and differences in socioeconomic development among regions of China all negatively influence the accessibility of cancer drugs (12). Accessibility of anticancer medicines also varies regionally, with accessibility in eastern regions of China expected to be greater than that in other regions owing to greater economic development (13). To date, most studies have reported on the accessibility of anticancer medicines in eastern China (14–16) but have rarely focused on that in northwestern provinces, the least developed regions of the country. Therefore, in the present study, we aimed to explore the accessibility of anticancer medicines in public hospitals of Northwestern China.

## 2. Materials and methods

### 2.1. Drug selection

We selected Shaanxi Province as the most representative region of Northwestern China. To ensure access to anticancer medicines, the government of Shaanxi implemented the policy of the Special Administration Drug List (SADL) in 2018. The SADL in Shaanxi mainly included nationally negotiated medicines and high-cost Class B medicines, both of which could be prescribed with reimbursement in outpatient and inpatient. The SADL was determined by the Shaanxi Provincial Healthcare Security Administration and updated annually. It was implemented by the “Three Designations” and “Dual-Channel” policy, which referred to designated physicians, public hospitals, and retail pharmacies. The number of medicines included in the SADL increased from 43 in 2018 to 126 in 2021. In 2021, the SADL included 95 nationally negotiated medicines and 31 Class B medicines, and the surveyed medicines in the present study were selected from the 2021 SADL.

To explore the continuous change in accessibility of these medicines, we selected 31 medicines that were commonly available in the SADL during the years 2018–2021 for our survey (Table 1). Among these, nine injectable medicines and 22 oral medicines were finally selected, with 11 Class B medicines and 20 nationally negotiated medicines. The Class B medicines were conventionally old drugs included in the national reimbursement drug list, and their drug costs were usually stable. In contrast, the nationally negotiated medicines were innovative drugs included in the NRDL for a short time to market.

**Table 1:** Characteristics of 31 surveyed anticancer medicines.

.	Generic name	Indications	Dosage Form	DDD/mg
1	Cetuximab	Colorectal cancer	Injection	60.7
2	Pegaspargase	Leukemia	Injection	303.6 IU
3	Nimotuzumab	Nasopharyngeal carcinoma	Injection	14.3
4	Recombinant Human Endostatin	Lung cancer	Injection	8.5
5	Bevacizumab	Colorectal cancer, lung cancer	Injection	50.0
6	Azacitidine	Myelodysplastic syndrome, leukemia	Injection	100.0
7	Rituximab	Lymphoma	Injection	115.9
8	Bortezomib	Myeloma, lymphoma	Injection	0.4
9	Pemetrexed	Lung cancer, alignant pleural mesothelioma	Injection	40.5
10	Osimertinib	Lung cancer	Oral	80.0
11	Anlotinib	Lung cancer, soft tissue sarcoma	Oral	8.0
12	Crizotinib	Lung cancer	Oral	500.0
13	Ceritinib	Lung cancer	Oral	450.0
14	Pazopanib	Renal cell carcinoma	Oral	800.0
15	Axitinib	Renal cell carcinoma	Oral	10.0
16	Regorafenib	Hepatocellular carcinoma, colorectal cancer, Gastrointestinal stromal tumor	Oral	120.0
17	Nilotinib	Leukemia	Oral	600.0
18	Ibrutinib	Lymphoma, leukemia, Waldenstrom’s macroglobulinemia	Oral	420.0
19	Vemurafenib	Melanoma	Oral	1920.0
20	Apatinib	Gastric adenocarcinoma or gastroesophageal junction adenocarcinoma	Oral	1250.0
21	Chidamide	Lymphoma	Oral	8.6
22	Everolimus	Renal cell carcinoma, endocrine tumor, lipoma, Astrocytoma	Oral	10.0
23	Erlotinib	Lung cancer	Oral	150.0
24	Sorafenib	Renal cell carcinoma, hepatocellular carcinoma, thyroid cancer	Oral	800.0
25	Afatinib	Lung cancer	Oral	40.0
26	Sunitinib	Renal cell carcinoma, gastrointestinal stromal tumor, endocrine tumor	Oral	33.0
27	Icotinib	Lung cancer	Oral	125.0
28	Lenalidomide	Myeloma	Oral	10.0
29	Gefitinib	Lung cancer	Oral	250.0
30	Imatinib	Leukemia, Gastrointestinal stromal tumor	Oral	400.0
31	Dasatinib	Leukemia	Oral	100.0

DDD, defined daily dose.

### 2.2. Sampling

There are 10 major cities in Shaanxi Province, and Xi’an is the capital city. Considering the geographic and economic differences among cities, Shaanxi Province can be divided into three regions: high-income regions (Yulin, Yan’an, and Xi’an), middle-income regions (Weinan, Xianyang, Tongchuan, and Baoji), and low-income regions (Hanzhong, Ankang, and Shangluo) (17).

The number of designated public hospitals varies among Shaanxi’s 10 cities, ranging from three in Hanzhong to 52 in Xi’an. Xi’an is the capital city with more than one-third of designated public hospitals in Shaanxi. Therefore, we considered Xi’an as a separate region in our study. Consequently, the designated public hospitals in this study were divided into four groups: those in the capital city (Xi’an), high-income regions (Yulin and Yan’an), middle-income regions (Weinan, Xianyang, Tongchuan, and Baoji), and low-income regions (Hanzhong, Ankang, and Shangluo), which have 52, 33, 46, and 15 designated public hospitals, respectively.

### 2.3. Data source

We collected data from all 146 designated public hospitals in Shaanxi Province. The procurement data of 31 anticancer medicines were extracted from the Shaanxi Drug Centralized Purchasing Platform, a provincial platform that provides hospital information and detailed drug procurement data for each hospital in Shaanxi. We extracted procurement data from January 1, 2019 to December 31, 2021. The data collected in our study included the month purchased, name of the purchasing medical institution, location of purchasing medical institution, amount purchased, quantity purchased, drug code, generic name, dosage forms, specifications, and conversion factor.

### 2.4. Primary outcomes

Three primary outcomes of anticancer medicines were applied in the present study, including availability, drug utilization, and affordability. The availability in each region was defined as the proportion of public hospitals with availability of these drugs to the total number of surveyed hospitals, calculated as follows:


$$\text{Availability}=\frac{\text{Number of hospitals with available anticancer medicines}}{\text{Total number of designated hospitals in each region}}\times 100\%$$

The drug utilization of anticancer medicines was evaluated using the amount purchased, defined daily doses (DDDs), and defined daily drug cost (DDDc). DDDs is an indicator developed by the World Health Organization (WHO) to evaluate the consumption and change trend of medicines. A higher DDD indicates higher use frequency of anticancer medicines. DDDs are calculated using the DDD, which is derived from the ATC/DDD Index 2022 published by the WHO (18). If there is no determined value for anticancer drugs in which the therapeutic dosage depends on the patient’s characteristics, we calculated the DDD for an adult with a body surface area of 1.7 m^2^ and weight of 70 kg (19). The value of the DDD of each anticancer medicine is displayed in Table 1. DDDc represents the daily expenditure for a certain medicine per one patient with cancer, which is calculated using the amount of the medicine purchased divided by the DDDs. The equations for calculating the DDDs and DDDc are as follows:


$$\text{DDDs}=\frac{\;\mathrm{Quantity of medicine purchased}\times \mathrm{conversion coefficient}\;}{DDD}$$

$$\text{DDDc}=\frac{\text{Amount of medicin purchased}}{\mathrm{DDDs}}$$

In the present study, the catastrophic health expenditure (CHE) was used to evaluate the affordability of anticancer medicines. When the annual OOP drug cost reached the threshold ≥40% of household expenditures, CHE occurred. Medicines were considered affordable when the OOP cost was below 40% of the household’s capacity to pay. Additionally, the affordability of anticancer medicines was analyzed for urban and rural households separately (20). The annual disposable incomes *per capita* in urban and rural regions were derived from the Shaanxi provincial Bureau of Statistics (21). We considered 30% to be the minimum reimbursement level of the SADL in Shaanxi in the base-case scenario (22). Therefore, anticancer medicines were considered affordable in our study if the annual OOP medicine costs were below CNY 28,878.40 and CNY 9,896.80; CNY 30,294.40 and CNY 10,652.80; and CNY 32,570.40 and CNY 11,796.00 for urban and rural residents in each year from 2019 to 2021, respectively.

### 2.5. Data analysis

The availability, amount purchased, DDDs, and DDDc are presented in descriptive statistics using mean and 95% confidence interval (CI), and the Wilcoxon rank-sum test and Friedman’s test were performed to compare their differences among different years, regions, dosage forms, and drug catalogs. The differences in affordability of anticancer medicines among different types of residents, dosage forms, and drug catalogs were analyzed by Wilcoxon rank-sum test. Spearman correlation analysis was applied to explore the correlation relationship among the primary outcomes. Data analyses were performed using Stata 16.0 (StataCorp LLC, College Station, TX, United States). Statistical tests were two-sided, with significance determined at *p* < 0.05.

## 3. Results

### 3.1. Availability

Thirty-one medicines were available in Shaanxi in 2021. The mean availability of the 31 surveyed medicines increased from 5.45% in 2019 to 14.72% in 2021, as shown in Table 2. The availability of anticancer medicines in Shaanxi Province was significantly different across regions (*p* < 0.001), and Xi’an had the highest mean availability (14.77%). The mean availability of nationally negotiated medicines was significantly lower than that of Class B medicines (8.72% vs. 12.85%, *p* = 0.048). The mean availability of injectable medicines was significantly higher than that of oral medicines (13.66% vs. 8.77%, *p* = 0.007). The mean availability of 27 medicines (87.1%) was lower than 20% and only one drug (gefitinib) had availability >30%.

**Table 2:** Associated factors of availability, purchasing amount, DDDs, and DDDc of anticancer medicines.

Variable		Availability (%)			Purchasing amount (million CNY)			DDDs			DDDc (CNY)		
		Mean	95% CI	*p*	Mean	95% CI	*p*	Mean	95% CI	*p*	Mean	95% CI	*p*
Year	2019	5.45	3.80–7.99	<0.001	6.51	2.69–10.33	0.007	225.50	124.15–326.86	0.008	551.15	400.30–763.70	<0.001
	2020	10.40	7.15–13.65		13.26	6.70–19.82		650.15	291.62–1008.67		490.29	330.46–650.12	
	2021	14.72	10.51–18.93		18.56	9.29–27.84		1019.50	527.06–1511.95		404.50	280.50–528.51	
Region	Capital city	14.77	11.41–18.14	<0.001	10.19	4.92–15.47	<0.001	279.81	127.94–431.67	<0.001	487.05	338.21–635.89	0.106
	High-income	6.61	4.27–8.95		0.38	0.12–0.63		12.40	3.08–21.73		488.92	328.79–649.05	
	Middle-income	9.77	6.07–13.47		0.86	0.42–1.29		38.33	2.75–73.91		475.63	325.53–625.72	
	Low-income	9.61	6.24–12.98		1.35	0.62–2.70		55.19	8.08–102.30		403.04	263.95–542.13	
Drug category	Nationally Negotiated Medicines	8.72	6.60–10.85	0.048	13.47	6.56–16.48	0.300	457.86	249.11–666.60	0.021	480.36	427.63–533.08	0.006
	Class B Drug List	12.85	8.95–16.76		11.52	7.84–19.09		947.83	499.42–1396.24		513.92	275.29–752.55	
Dosage form	Injection	13.66	9.56–17.75	0.007	20.52	10.67–30.38	0.002	519.21	251.21–787.21	0.379	761.89	513.24–1010.54	0.003
	Oral dosage form	8.77	6.62–10.93		9.61	5.76–13.45		677.74	400.96–954.52		381.96	321.47–442.46	

CNY, Chinese Yuan; DDDs, defined daily doses; DDDc, defined daily drug cost; CI, confidence interval.

The availability of each medicine increased significantly during 2019–2021 (*p* < 0.001). The mean availability varied between 0.96% for vemurafenib and 32% for gefitinib. Gefitinib continued to have the highest availability in these consecutive 3 years (Table 3), whereas the lowest availability was for vemurafenib in 2021 (1.18%). Azacitidine had the highest annual increase in availability during 2019–2021, which reached 267.39%.

**Table 3:** Availability, purchased amount, DDDs, and DDDc of each anticancer medicine.

No.	Generic name	Availability (%)				Amount purchased (million CNY)				DDDs				DDDc (CNY)			
		2019	2020	2021	Mean	2019	2020	2021	Mean	2019	2020	2021	Mean	2019	2020	2021	Mean
1	Cetuximab	7.25	10.46	13.82	10.51	8.67	18.48	41.89	23.01	136.88	187.56	269.99	198.14	786.19	812.21	742.85	780.42
2	Osimertinib	9.23	19.13	29.98	19.45	17.81	74.44	147.65	79.97	503.00	1423.00	3571.33	1832.44	510.00	507.39	238.39	418.59
3	Anlotinib	16.08	17.31	28.34	20.58	4.14	71.86	149.71	75.23	736.87	2453.91	3739.61	2310.13	300.94	308.51	246.94	285.46
4	Crizotinib	3.63	8.00	9.37	7.00	5.54	12.44	26.48	14.82	161.20	230.00	246.27	212.49	520.37	521.52	468.57	503.49
5	Ceritinib	0.42	1.27	3.26	1.65	0.03	1.15	1.87	1.01	0.56	16.11	18.89	11.85	297.00	297.00	423.50	542.33
6	Pazopanib	1.51	4.18	7.46	4.38	1.01	6.09	11.85	6.32	43.24	96.76	207.36	115.79	640.00	640.00	640.00	640.00
7	Axitinib	0.97	3.21	5.14	3.11	1.40	5.90	13.29	6.86	43.24	96.76	207.36	115.79	414.00	413.94	396.29	408.08
8	Regorafenib	3.84	5.83	10.17	6.62	5.75	24.58	50.52	26.95	133.99	281.35	467.08	294.14	588.00	597.39	531.41	572.27
9	Nilotinib	1.61	4.27	5.14	3.67	0.64	3.70	7.55	3.96	27.33	109.89	165.67	100.96	302.35	301.29	295.39	298.77
10	Ibrutinib	3.59	7.04	6.02	5.55	0.90	6.04	12.49	6.47	17.67	62.67	119.67	66.67	567.00	567.00	516.54	550.18
11	Vemurafenib	0.42	1.27	1.18	0.96	0.10	0.33	0.50	0.31	1.24	3.19	1.01	1.81	672.00	896.00	762.14	843.94
12	Pegaspargase	4.22	4.90	5.33	4.82	0.87	3.37	7.90	4.05	41.31	98.55	179.53	106.46	241.24	241.24	241.24	241.24
13	Azacitidine	0.97	5.07	10.67	5.57	0.05	4.31	8.15	4.17	0.37	21.31	65.79	29.15	1793.50	1645.75	657.54	1368.31
14	Afatinib	7.34	11.52	12.13	10.33	2.54	9.82	19.93	10.76	152.17	379.52	550.61	360.77	208.14	207.69	202.91	206.25
15	Sunitinib	3.63	5.11	9.57	6.10	3.38	10.44	22.77	12.20	99.11	173.00	263.86	178.65	409.20	383.63	409.20	400.68
16	Icotinib	6.92	6.92	12.75	8.86	6.11	12.37	32.00	16.82	1071.00	1066.80	2344.77	1494.19	63.84	63.83	64.05	63.91
17	Nimotuzumab	6.45	8.93	9.57	8.32	2.62	10.90	24.44	12.65	69.67	192.33	321.09	194.36	485.86	410.12	410.12	435.37
18	Apatinib	5.92	15.52	18.77	13.40	2.49	10.35	20.78	11.20	42.07	110.20	153.42	101.89	656.30	574.74	575.00	602.01
19	Recombinant Human Endostatin	9.54	15.04	18.89	14.49	10.60	16.57	40.65	22.61	401.84	486.35	709.55	532.58	357.00	277.67	277.67	304.11
20	Chidamide	1.61	2.45	2.88	2.31	0.48	1.63	3.43	1.85	10.27	20.23	24.89	18.46	604.91	587.90	587.90	598.15
21	Everolimus	0.85	2.15	3.00	2.00	0.07	0.46	1.00	0.51	4.67	11.67	21.58	12.64	287.12	260.00	261.64	269.18
22	Erlotinib	3.75	8.83	9.05	7.21	1.40	2.01	4.97	2.79	140.62	238.47	301.78	226.96	181.83	80.18	78.36	113.46
23	Sorafenib	5.35	10.74	20.88	12.32	42.88	93.39	179.02	105.10	642.50	1788.50	1557.42	1329.47	762.48	373.23	261.13	465.61
24	Rituximab	10.01	17.71	27.47	18.40	25.73	68.38	151.34	81.82	142.46	266.19	370.93	259.86	2468.18	2013.27	1840.53	2107.33
25	Lenalidomide	3.26	7.13	9.94	6.78	0.72	7.52	16.31	8.19	124.25	659.17	1290.57	691.33	76.42	78.32	76.64	77.97
26	Bortezomib	2.36	6.28	17.19	8.61	4.77	20.84	41.69	22.43	90.49	218.24	1058.02	455.58	660.86	816.19	337.38	605.36
27	Gefitinib	16.32	38.26	41.43	32.00	3.40	22.06	41.56	22.34	654.11	4293.78	4558.22	3168.70	156.82	51.59	52.71	79.26
28	Imatinib	8.35	19.54	23.53	17.14	2.58	12.11	27.01	13.90	511.83	2409.23	3750.07	2223.71	112.82	61.69	59.91	78.25
29	Dasatinib	0.00	0.00	4.59	1.53	0.00	0.00	1.11	0.37	0.78	0.00	123.74	41.51	106.26	/	94.06	100.16
30	Pemetrexed	13.45	27.31	37.42	26.06	9.39	54.33	110.97	58.23	467.86	1537.96	2562.14	1522.65	446.31	235.11	224.01	280.41
31	Bevacizumab	10.13	26.99	41.27	26.13	35.68	114.77	259.01	136.48	518.02	1221.84	2382.40	1374.09	960.43	669.33	573.59	734.45

DDDs, defined daily doses; DDDc, defined daily drug cost; CNY, Chinese Yuan.

### 3.2. Drug utilization

The total purchased amount rose from CNY 201.74 million in 2019 to CNY 1,477.83 million in 2021. The amount purchased in Xi’an was significantly higher than that in other regions (*p* < 0.001), as shown in Table 2. The mean purchased amount of nationally negotiated medicines showed no significant difference compared with that of Class B medicines (CNY 27.11 million vs. CNY 22.83 million, *p* = 0.300). The mean purchased amount of injectable medicines was significantly higher than that of oral medicines (CNY 40.61 million vs. CNY 19.45 million, *p* = 0.002). The purchased amount of each medicine increased significantly each year (*p* = 0.007). The mean purchased amount varied from CNY 0.31 million for vemurafenib to CNY 136.48 million for bevacizumab (Table 3). Sorafenib had the highest purchased amount (CNY 42.88 million) in 2019 and bevacizumab had the highest in 2020 and 2021 (CNY 114.77 million and CNY 259.01 million, respectively). There was a positive correlation between availability and purchased amount (*r*_s_ = 0.862, *p* < 0.001).

The mean DDDs of the 31 surveyed medicines rose from 225.50 to 1019.50 during 2019–2021. The mean DDDs of anticancer medicines in Xi’an was significantly higher than that in other regions (*p* < 0.001), as shown in Table 2. The mean DDDs of Class B medicines was significantly higher than that of nationally negotiated medicines (*p* = 0.021), and there was no significant difference in DDDs between different dosage forms (*p =* 0.379). The DDDs of each medicine increased significantly from 2019 to 2021 (*p* = 0.008), and the mean DDDs varied between 1.81 for vemurafenib and 3168.70 for gefitinib (Table 3). Icotinib had the highest DDDs (1071.00) in 2019 and gefitinib had the highest DDDs in 2020 and 2021 (4293.78 and 4558.22, respectively). There was a positive correlation between availability and DDDs (*r*_s_ = 0.869, *p* < 0.001).

The yearly mean DDDc of the 31 medicines decreased from CNY 551.15 to CNY 404.50 during 2019–2021 (Table 2). There was no significant difference in DDDc among regions (*p* = 0.106). The mean DDDc of nationally negotiated medicines was significantly higher than that of Class B medicines (*p* = 0.006). The mean DDDc of injectable medicines was significantly higher than that of oral medicines (*p* = 0.003). The DDDc of each medicine decreased significantly during the 3 years (*p* < 0.001), and the mean DDDc varied between CNY 63.91 for icotinib and CNY 2,107.33 for rituximab, as shown in Table 3. There was no significant correlation between availability and DDDc (*r*_s_ = −0.096, *p* = 0.608).

### 3.3. Affordability

**Figure 1:**
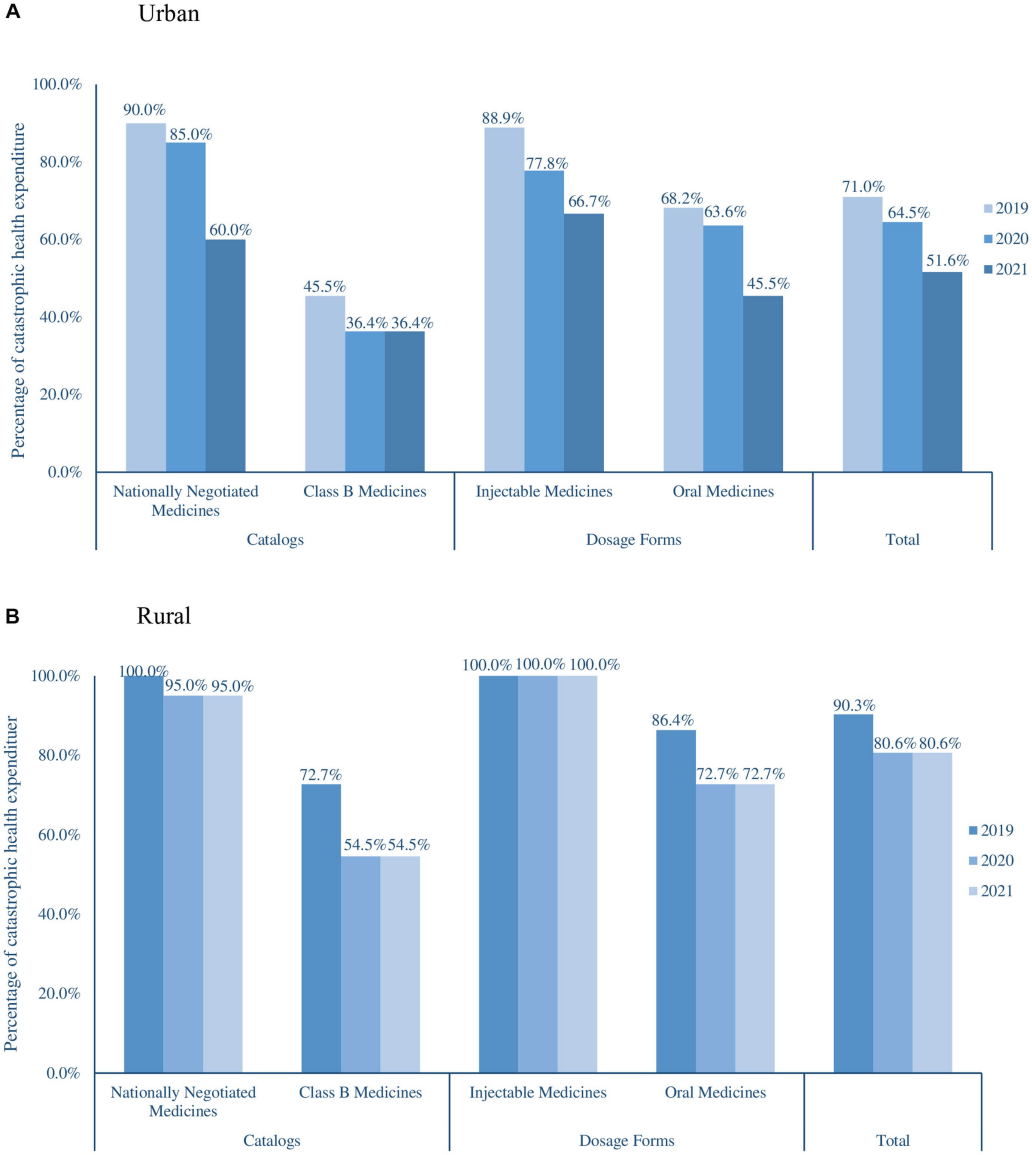
Affordability of anticancer medicines in urban **(A)** and rural areas **(B)**.

The percentage of CHE decreased annually from 2019 to 2021. The percentage of CHE decreased from 71.0 and 90.30% in 2019 to 51.65 and 80.60% in 2021 for urban and rural residents, respectively (Figure 1), the affordability of anticancer medicines of urban residents was significantly higher than that of rural residents (*p* < 0.001). In both urban and rural areas, the affordability of nationally negotiated medicines was significantly lower than that of Class B medicines (*p* = 0.032), and the affordability of injectable medicines was lower than that of oral medicines (*p* = 0.124).

**Figure 2:**
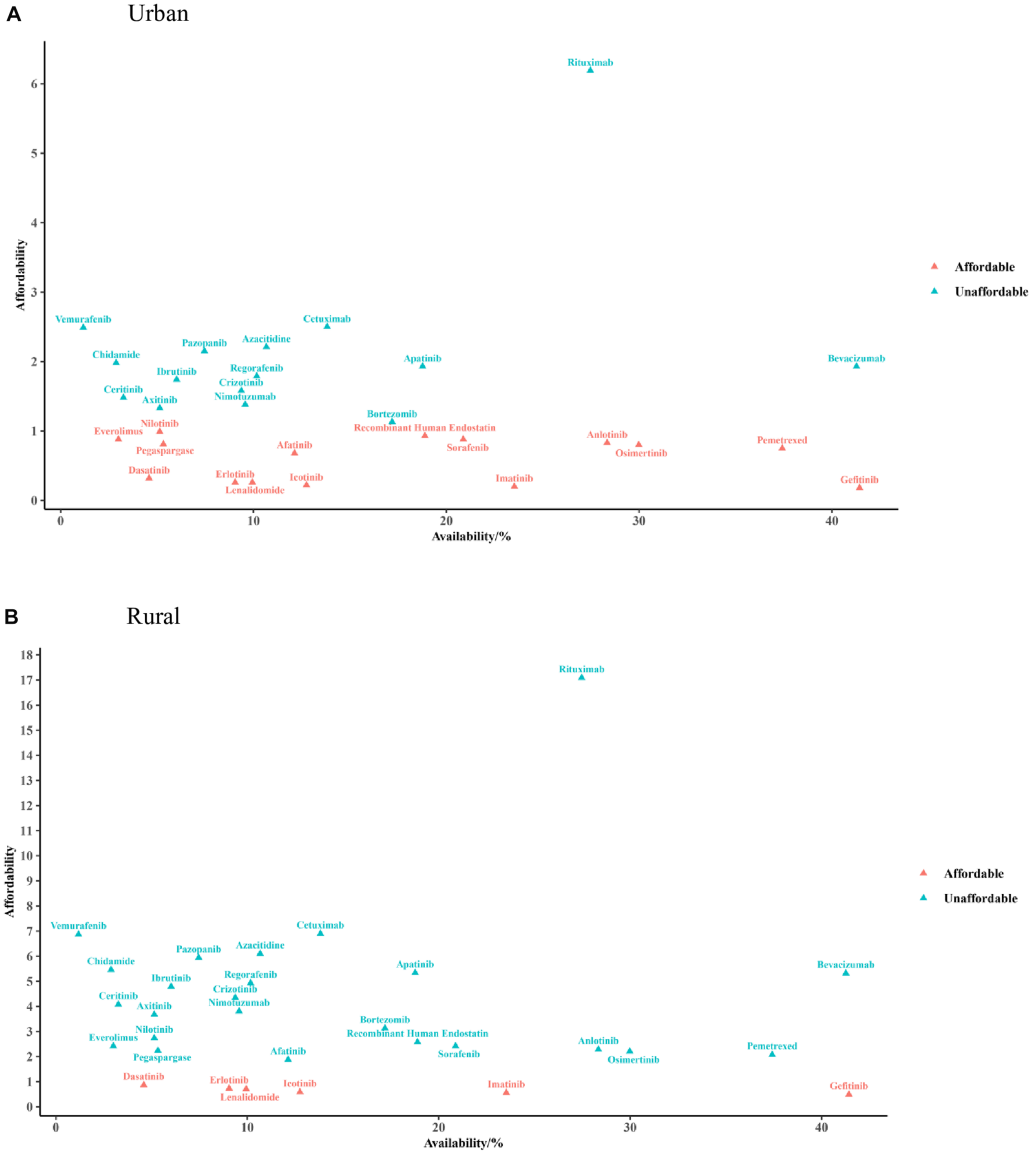
Cross-over analysis of availability and affordability in urban **(A)** and rural areas **(B)**. The triangular dots indicate the availability of each medicine in public hospitals (X-axis) and the times of catastrophic health expenditure for affordability (Y-axis) in 2021.

Figure 2 shows the results of a comprehensive analysis between the availability and affordability of anticancer medicines in 2021. For urban residents, three (9.68%) medicines had good availability (≥30%) with high affordability, among which gefitinib concurrently had the best availability and affordability (0.18-fold over the threshold). Fourteen (45.16%) medicines had poor availability with low affordability, among which vemurafenib had the poorest availability (1.18%) and rituximab had the poorest affordability (6.19-fold over the threshold). For rural residents, only gefitinib simultaneously had superior availability and affordability (0.49-fold over the threshold), and five (16.13%) affordable medicines were poorly available. Twenty-one (67.74%) medicines had poor affordability and poor availability; rituximab and cetuximab had the poorest affordability (6.90- and 17.09-fold over the threshold, respectively).

## 4. Discussion

To our knowledge, this was the first study to assess the accessibility of anticancer medicines in Northwestern China. We incorporated a total of 146 public hospitals in 10 cities of Shaanxi in this study and examined drug availability, utilization, and affordability to comprehensively evaluate drug accessibility. Our results showed that the availability, utilization, and affordability of anticancer medicines all improved significantly in the public hospitals surveyed during 2019–2021.

As for availability, our results showed a significant uptrend, with an average annual growth rate varying between 12.37 and 267.39% of 31 surveyed medicines. In 2019, the availability of all 31 medicines was below 20.0%; by 2021, the availability of eight (25.81%) anticancer medicines was over 20%, and the availability for the leading two drugs reached 41.43% for gefitinib and 41.27% for bevacizumab. Similar findings were reported in a study from Nanjing, with mean availability of gefitinib, bevacizumab, and recombinant human endostatin increasing from <30% in 2017 to 60.33% in 2020 (14). Incorporating more innovative cancer drugs into the NRDL combined with reimbursement policies, could improve the availability of anticancer medicines (14, 15). However, the overall availability of anticancer medicines was still unsatisfactory, with less than half of these drugs available in public hospitals in Shaanxi. Similarly, a study from Sichuan reported that the availability of 95.88% of investigated anticancer medicines was less than 30%, among which 78.35% were unavailable in 2020 in all tertiary public hospitals (23). The reasons for the low availability of anticancer drugs in public hospitals might be multifactorial, involving insufficient funding of public hospitals, a lack of incentives to maintain sufficient stocks, and inefficient procurement systems (24, 25). Therefore, multiple measures could be combined to further increase the drug availability in China, such as priority review and conditional approval for innovative cancer drugs (26, 27), as well as expanding funding and improving key performance indicators for public hospitals.

Our findings suggested that the regional disparity in drug availability is considerable in Shaanxi, with high-income regions having greater availability and differences across regions at the national level (13). This result is supported by findings in Hubei Province, where the mean availability of gefitinib in 2019 was 23.81%, which was higher than that of our study at 16.32% (28). Our findings could also be attributed to differences in socioeconomic development and different capacities in the governance provided (29). The significant difference in dosage forms might be explained by the fact that injectable drugs can only be used in hospitals whereas oral dosage forms are available in both public hospitals and designated retail pharmacies. We also observed some variations in the availability of each drug. First, drugs for cancers with higher morbidity and mortality in China had greater availability, which reinforced reports from previous studies (12, 30). Lung cancer has the highest incidence in China; therefore, drugs for treating lung cancer, such as gefitinib and pemetrexed, had greater availability. Additionally, medicines that were included early in the NRDL had more availability (28). Class B medicines usually require a longer time for market and are included in the NRDL earlier whereas nationally negotiated medicines are innovative drugs with a short time until they become available on the market. This might explain the significant difference in availability between different catalogs.

Our findings indicated a significant increase in procurement volumes and DDDs and a significant decrease in DDDc of the surveyed anticancer medicines during 2019–2021. Including anticancer medicines in medical insurance with partial reimbursement and national pricing negotiations could boost the use and lower the costs of anticancer medicines (27, 31), with improvement in patients’ access to these drugs (32). Additionally, the DDDc of anticancer medicines in our study did not show a significant difference among regions. Similar results have been reported previously, which confirms the effectiveness of national drug pricing negotiations in unifying medicine prices across a country (12).

We found that the affordability of anticancer medicines increased annually, but the expenditure was still unreasonable for most families owing to high prices. Annual OOP spending on the 31 anticancer medicines was 0.18- to 6.19-fold and 0.49- to 17.09-fold the threshold 40% of annual household income for urban and rural residents in Shaanxi, respectively. In Hangzhou, targeted payments for anticancer medicines were 0.6- to 2.1-fold and 1.8- to 4.4-fold the average annual disposable income *per capita* for urban and rural residents after government health insurance coverage inclusion (16). In Mexico, most anticancer medicines are also unaffordable for patients, with the median daily cost of patented cancer medicines equaling wages for 30.17 days (33). Price is a vital factor hindering access to anticancer medicines. It is therefore imperative that the Chinese government reasonably control the price of cancer drugs *via* national drug price negotiation with dynamic updates of the NRDL, as well as publication of a volume-based price list (34). Our findings showed that urban residents usually had better affordability of any anticancer medicines than rural residents. These results were similar to research in Jiangsu (35) and Sichuan (23). In rural areas of China, health financing structural imbalance and funding shortages may hinder drug affordability. Therefore, further investment in health resources, strengthening medical insurance for major diseases, and medical assistance for low-income people are urgently needed to ensure equal access to anticancer medicines (36, 37). Additionally, precision policies to promote the accessibility of anticancer medicines are warranted. Due to the imbalance of diagnosis and treatment capabilities of cancer in different regions and hospitals, the local government needs to allocate resources to improve the access of anticancer medicines to institutions that in real need.

There were several limitations in the present study. First, owing to limited data sources, only Shaanxi was selected as the study province, and the accessibility of anticancer medicines in other provinces of Northwestern China might be overestimated owing to economic development levels that are even lower than those of Shaanxi. Second, a certain drug was considered available if it was stocked on any day in a quarter; however, drug supply continuity and shortages during the study period were ambiguous, which might lead to overestimation regarding the availability of anticancer medicines. Third, subgroup analysis for different types of public hospitals was not performed, and we could not further compare the drug accessibility levels in tertiary and secondary hospitals.

## 5. Conclusion

The accessibility of anticancer medicines increased annually in public hospitals in Northwestern China during 2019–2021. Additional incentive and collaborative policies are warranted to further improve the accessibility level of these drugs.

## Data availability statement

The raw data supporting the conclusions of this article will be made available by the authors, without undue reservation.

## Author contributions

YM and MJ designed the study. YM, JP, LF, and XS collected and analyzed the data. YM, XY, and MJ drafted the initial manuscript. MJ revised the manuscript. All authors contributed to the article and approved the submitted version.

## Funding

This work was supported by the Natural Science Foundation of Shaanxi Province [grant number 2023-YBSF-374], and the Philosophy Social Science Fund Youth Program of Shaanxi Province [grant number 2023QN0002].

## Conflict of interest

The authors declare that the research was conducted in the absence of any commercial or financial relationships that could be construed as a potential conflict of interest.

## Publisher’s note

All claims expressed in this article are solely those of the authors and do not necessarily represent those of their affiliated organizations, or those of the publisher, the editors and the reviewers. Any product that may be evaluated in this article, or claim that may be made by its manufacturer, is not guaranteed or endorsed by the publisher.
